# Multilevel analysis of individual- and community-level determinants of birth certification of children under-5 years in Nigeria: evidence from a household survey

**DOI:** 10.1186/s12889-022-14786-2

**Published:** 2022-12-14

**Authors:** Uchechi Shirley Anaduaka

**Affiliations:** 1 Department of Economics, University of Nigeria, Nsukka, Enugu State, 410001 Nigeria; 2 Department of Economics, Lingnan University, Tuen Mun, Hong Kong SAR, China

**Keywords:** Civil registration and vital statistics *(*CRVS), Birth certification, Multilevel analysis, Complex sampling design, Nigeria

## Abstract

Promoting birth certification is central to achieving legal identity for all - target 16.9 of the 2030 Sustainable Development Goals. Nigeria is not on track to achieve this goal with its low coverage of birth certification (BC). This study is aimed at identifying patterns of BC and its associated individual- and community-level factors, using pooled cross-sectional data from three rounds (2008, 2013, and 2018) of the nationally representative Nigerian Demographic and Health Survey. A weighted sample of 66,630 children aged 0–4 years was included, and a two-level multilevel logistic model which accommodates the hierarchical nature of the data was employed. Of the total sample, 17.1% [95% CI: 16.3–17.9] were reported to be certified. Zamfara state (2.3, 95% CI: 0.93–3.73) and the Federal Capital Territory (36.24, 95% CI: 31.16–41.31) reported the lowest and the highest BC rates. Children with an SBA [AOR = 1.283, 95% CI: 1.164–1.413] and with at least one vaccination [AOR = 1.494, 95% CI: 1.328–1.681] had higher odds of BC. The AOR for mothers with at least one prenatal visit was 1.468 [95% CI: 1.271–1.695], and those aged 30–34 years at the time of birth [AOR = 1.479, 95% CI: 1.236–1.772] had the highest odds. Further, the odds of BC increased the most for mothers [AOR = 1.559, 95% CI: 1.329–1.829] and fathers [AOR = 1.394, 95% CI: 1.211–1.605] who were tertiary-educated. In addition, children in middle-income [AOR = 1.430, 95% CI: 1.197–1.707] or rich wealth HHs [AOR = 1.776, 95% CI: 1.455–2.169] or those whose families had bank accounts [AOR = 1.315, 95% CI: 1.187–1.456] had higher odds. Living in non-poor and within close proximity to a registration center (RC) act as protective factors for BC, while living in poor communities [AOR = 0.613, 95% CI: 0.486–0.774] and more than 10kms from an RC reduce the odds of BC [AOR = 0.466, 95% CI: 0.377–0.576]. The study identified several protective and risk factors which policymakers can adopt as strategic areas for universal birth certification. National and sub-national programs should integrate non-formal institutions as well as target child and maternal utilization of healthcare services to promote BC in Nigeria.

**Supplementary Information:**

The online version contains supplementary material available at 10.1186/s12889-022-14786-2.

## Background

In the past decade, child protection indicators such as birth registration (BR)(^1^ BR is defined as “the continuous, permanent, and universal recording within the civil registry, of the occurrence and characteristics of births in accordance with the legal requirements of a country” [1].) have been incorporated into the development goals agenda [2]. For example, target 16.9 of the Sustainable Development Goals (SDG) suggests a *legal identity for all* by 2030. The United Nations Convention on the Rights of the Child (UNCRC) notes that BR is a child’s ‘first right’ as it provides the legal ‘membership card’ to participate in society [3, 4]. In addition, it is considered critical to improvements in human capital outcomes - utilization of healthcare services and educational attainment (school enrolment, progression and completion [5]. Without being registered, a child will be born and die without any trace of their existence documented officially. This term is known as the *scandal of invisibility –* being unseen, uncountable, and hence uncounted [6, 7].

Universal BR is a public good that is used to monitor the progress of development goals and the functioning of the rule of law in modern societies [1, 8]. As a proof, registered children are issued a *birth certificate,* which documents their right to an identity and ensures that their human rights are not overlooked when violated or abused [5, 9, 10]. In some countries, birth certificates are required for enrolment, taking tests and examinations, scholarship applications and graduation for primary and secondary schools [11]. This valid proof of age can thus mitigate long-term risks by making it easier to claim rights and/or privileges in the future, e.g., formal labour market participation, civil marriage registration, bank account ownership, owning/inheriting land and housing, access to social welfare programs (e.g. scholarships, food assistance, insurance and pensions), voting and obtaining a passport for international travel [12–15].

In Nigeria, 70% of children under five years of age are unregistered, and 83% of them do not have birth certificates, according to a recent household survey [16]. The rates of non-documentation of births in Nigeria are among the world’s highest, and rank higher than those in Sub-Saharan Africa (59%) and West and Central Africa (55%) [17]. To address the ‘scandal of invisibility’, it is important to identify the protective and risk factors of birth registration to facilitate well-targeted policies. For example, some children may not be registered due to the low number of BR centres in their community (low supply), while others may be due to being born into low-income families or poorly educated parents (low demand).

Extant studies suggest that the proximate determinants of child survival are most likely related to the underlying factors that affect a child’s probability of having their births registered or certified [18]. Some of the identified socioeconomic and demographic (SED) factors deemed important for BR include *the gender of the child* (Nigeria) [19]; *utilization of perinatal health services* (Edo, Nigeria; Nusa Tenggara, Indonesia) [20, 21]; *mother’s age at child’s birth* (Ghana; Western Australia; Bolivia, Brazil, Colombia, the Dominican Republic, Peru, and Nicaragua) [22–24]; *maternal health-seeking behavior* (Bauchi and Cross-River, Nigeria) [25]; *parent’s literacy and education* (Lombok, Indonesia; Ghana) [26, 27]; *wealth* (Indonesia; Dominican Republic) [6, 28]; and *geographical location* (Bolivia, the Dominican Republic, and Peru) [29]. It is important to note that most of the existing studies on the determinants of birth certification are beyond the Nigerian context. Analysing how the identified determinants or factors play out in the Nigerian setting therefore makes an important contribution.

The objective of the current study is two-fold and aims to provide answers to two questions: (1) What is the pattern and coverage of birth certification and (2) what individual and community factors are associated with birth certification for children under-five years of age? To achieve the stated objectives, the study employs descriptive statistics and multilevel regression models to analyse a pooled sample of data of children under-five years from the last three rounds of the nationally representative NDHS data. The adoption of the multilevel regression analysis provides insight into what level of factors are most critical in driving BC in Nigeria. The study makes two key contributions to the literature. First, it adopts birth certification (BC) instead of BR (^2^ Birth certification and registration rates may differ for several reasons. First, parents may begin but not complete the registration process. This may be due to issues with payment of fees. While the Nigerian law states that the birth registration should be free at least for newborn children, some registration offices charge fees for this. Another reason could be linked to the fact that some mothers mistake the attestation of a live birth for the birth certification. Finally, some parents could have lost or have not picked up the birth certificate and so could not provide it at the time of the interview. These reasons are currently speculative and would need further research as evidence) because the latter is based on the parent/caregiver report and the outcome is open to bias. Second, the study captures supply-side factors e.g., the availability of civil registration centres, which have been missing in earlier studies. The lack of an enabling environment for birth registration within or close to a community can increase financial and opportunity costs, and hinder the efforts of parents/caregivers to register and certify their children’s births [29, 30]. Third, among the existing studies on Nigeria, none analysed the SED characteristics using different waves of the Nigerian Demographic & Health Survey (NDHS) while simultaneously accounting for the hierarchical structure of the data. Pooling data helps increase the sample size, and the enhanced statistical power allows for analyzing the factors associated with BC for comparable children across communities. In the NDHS data, individuals are nested within a cluster/community, and their characteristics may be similar to those living in the same cluster/community compared to the rest of Nigeria. Adjusting for the hierarchical nature of the data provides more realistic standard errors and estimates, as multilevel analysis explicitly models the correlation of responses at the group-level. In addition, it avoids relying on the assumption of independence of observations within the same survey cluster and equal variance across clusters [31]. The findings from the study will prove important for programs and policies targeted at improving BR and BC in Nigeria.

## Methods

### Study setting

The current study adopts multilevel logistic and poisson regression analyses to assess the correlates of birth certification (BC) in Nigeria. Nigeria, Africa’s populous country is divided into six geo-political regions (North-Central (NC), North-East (NE), North-West (NW), South-East (SE), South-South (SS) and South-West (SW)), which house the 36 self-governing states and the Federal Capital Territory (FCT) (see Additional file 1 for the list of states by their region).

The country is a viable study setting given the low prevalence of BC coupled with significant within country variations in SED characteristics which may create lopsided rates in birth certifications.(^3^ It also has significant cultural differences with more than 300 ethnic and three main religious groups.) (^4^ Three ethnicities (Hausa/Fulani, Igbo and Yoruba) dominate the cultural sphere: the Hausa/Fulani primarily reside in the north, the Igbo and Yoruba ethnicities reside in the SE and SW, respectively. Additionally, the north and south are divided across religious lines, with Islam primarily in the north and Christianity in the south.) For instance, the median years of schooling for women ranges from zero in the NE and NW to 8.2 years in the South West [16]. If maternal education is a key determinant of BC, as is the case with other indicators of social and economic development, this may create disparities in the patterns of BC between the northern and southern parts of Nigeria. It is crucial that an effective civil registration and vital statistics (CRVS) system that accommodates such variation in SED characteristics to drive BC and provide credible vital statistics is in place. In Nigeria, the responsibility to establish an effective CRVS system falls on the National Population Commission (NPC) established in 1988 by the Federal Government of Nigeria. The NPC is charged with two core responsibilities: carrying out civil registration and undertaking periodic enumeration of the population through sample surveys and censuses. Since 2003, following the introduction of the Child Rights Act, it became compulsory for every child’s birth to be registered within 60 days of birth [20]. Consequently, the NPC set a target of Universal Birth Registration (100%) by 2015. However, as of 2018, this mandate has fallen below par, as less than 43% of children have their births registered, and only 22% have their births certified [16].

### Data source, sample and design

The study is a secondary analysis of the nationally representative DHS (Demographic and Health Survey), a repeated cross-sectional standardized survey implemented in Nigeria, and focused on the demographic and health characteristics of Nigerian households (HHs).(^5^The data was collected by the Macro International Inc., Calverton, Maryland in cooperation with the U. S. Agency for International Development (USAID), the NPC, and the Federal Ministry of Health.)(^6^ The response rates are generally high. For instance, in the 2008 round, the response rate was 98% and 97% for HHs and women, respectively.)^.^ In addition to information on the child’s BC status, the survey collects key information on the socioeconomic, demographic and health information concerning women, men, children, HHs and the survey communities. The eligible HHs were selected using a multistage stratified random cluster sampling technique based on the 2006 census, with considerations for urban/rural areas [16]. First, clusters are drawn from an official list of the census enumeration areas (EAs), which usually correspond to small villages or blocks within larger villages or cities. Second, HHs are randomly drawn from the list of clusters. Finally, every eligible woman (aged 15–49 years) in the selected HH is interviewed and asked to provide information on herself, her children and spouse/partner (if available). The present study pools the 2008, 2013 and 2018 waves given that the outcome variable (BC) is available only in these years. A total of 88,644 children aged 0–4 years (0–59 months) had information on whether their births were certified. However, the final sample for the study is restricted to single-birth children born to mothers in a union (married or cohabiting) and co-resident with both parents at the time of the survey to allow for comparable features to be assessed. In addition, observations without GPS coordinates are dropped, as it was not possible to compute distance variables (to registration centres and roads) (see Additional File 1 for more details). Following the sample restrictions and exclusions of unusable records (those observations missing key SED variables), 66,630 children born to 46,672 mothers from 3127 communities within 37 states during the 2008–2018 period were deemed suitable for the analysis.

## Measures

### Outcome variable

The outcome variable for the current study is birth certification (BC). Studies have shown that there is a potential of inducing recall bias when using self-reported variables for retrospective information [32]. Therefore, this study employed BC as a more robust definition of BR rather than relying on the parent/caregiver report of whether the child is registered. BC was computed from responses to the following question: ‘*Does (NAME) have a birth certificate?’,* and coded as: 1 = Registered and has certificate, 2 = Registered, but no certificate seen, 3 = Not registered, 8 = Don’t Know. For the basis of this analysis, a dichotomous variable is generated with Yes = 1 if the child is registered and has a birth certificate and 0 otherwise.

### Independent variables

To accommodate the hierarchical nature of the NDHS data, two levels (individual and community) of SED factors with potential effects on BC were considered for analysis.. At the individual level (level one), we considered the characteristics of the children, their parents and the household in which they reside.

#### Child variables

Seven variables were included to capture the child’s demographic characteristics: gender (0 = female, 1 = male), age (months, 1 = 0–11, 2 = 12–23, 3 = 24–35, 4 = 36–47, 5 = 48–59), birth order among children of the same mother (1 = 1st child, 2 = 2nd child, 3 = 3rd child, 4 = 4th or higher order birth), reported size-at-birth (1 = small, 2 = average, 3 = large)(^7^ The size at birth is used as a proxy for the child’s birth weight as the latter is not available for most of the children in the sample. This indicator is commonly used in the literature and reported as a good measure for the child’s birth weight [33].

), birth interval (years, 0 =≤2.5 years, 1 = >2.5 years), skilled birth attendant (SBA, 0 = No, 1 = Yes), and vaccination status (0 = 0, 1 = 1+).

#### Parent-related variables

The eight variables employed to describe the mother’s characteristics include age at birth (years, 1 = <20 , 2 = 20–24, 3 = 25–29, 4 = 30–34, 5 = 35+), education (1 = None, 2 = Primary, 3 = Secondary, 4 = Tertiary), prenatal visits (0 = 0, 1 = 1+), polygynous (0 = No, 1 = Yes), occupation (1 = low skill, 2 = medium skill, 3 = high skill, 4 = other), decision-maker (0 = No, 1 = Yes), access to mass media (0 = No, 1 = Yes) and lost 2+ children (0 = No, 1 = Yes). For the father, three variables were employed: age (years, 1 <25 years, 2 = 25–34, 3 = 35–44, 4 = 45–54, 5 = 55+)^8^, education (1 = None, 2 = Primary, 3 = Secondary, 4 = Tertiary), and occupation (1 = low skill, 2 = medium skill, 3 = high skill, 4 = other).(^8^,Collapsing the child’s age, mother’s age at birth and the father’s age into cohorts allows the possibility of checking the existence of a non-linear relationship.)(^9^ The analysis utilizes a stock measure - the number of years of schooling reported for the mother and father separately - to capture the level of human capital in the household [16].)

#### Household-related variables

Four variables were included in the analysis: Owns a bank account (0 = No, 1 = Yes), wealth index (1 = poor, 2 = average, 3 = rich); religion (1 = Islam, 2 = Christianity, 3 = other), and ethnicity (1 = Hausa/Fulani(^10^ These ethnicities are grouped on the basis that they speak a common language or dialect, share a common sense of identity, cohesion and history; or have a single set of customs and behavioural norms e.g. marriage, clothing, diet, and taboos [34].), 2 = Igbo, 3 = Yoruba, 4 = Others). Given that the NDHS does not collect income or consumption data, the HH’s wealth status is based on the wealth index provided in the dataset. This index is constructed as a linear combination using the principal component analysis of the HH’s ownership of selected assets [35, 36]. The computed weights make more sense when HHs are distributed into one of five bins, ranging from *one* for the poorest fifth to *five* for the wealthiest fifth.(^11^ For a detailed discussion on the use of asset indices to capture the wealth status of HHs, see [35, 36]. For a more detailed description on how the wealth index is constructed, see [37].)

#### Community-related variables

The community-level (level two) characteristics were measured at the level of the geographic ‘cluster’ in which the HH was surveyed. Nine variables were included in the analysis: place of residence (0 = urban, 1 = rural), distance to registration centers (1≦5 kms, 2 = 5–9.99 kms, 3 = 10+ kms), distance to the nearest road (1≦5 kms, 2 = 5–9.99 kms, 3 = 10+ kms), altitude (meters above sea level; 1 = low (< 323), 2 = medium (323–449), 3 = high (> 449)), proportion of poor HHs (cutoff using the median value; 0≦0.4375, 1= > 0.4375), proportion of children who have died in the community (cutoff using the median value; 0≦0.54, 1 = at least 0.54), and the region of residence (1 = NC, 2 = NE, 3 = NW, 4 = SE, 5 = SS, 6 = SW). The distance to the nearest registration center and road, and the altitude are indicators of geographic accessibility. A detailed explanation on the coding and definitions of the explanatory variables is provided in the supplementary data file (see Additional file 1).

## Statistical analysis

First, univariate analysis using proportions was employed to describe the characteristics of the sample. Second, a bivariate cross-tabulation chi-squared test was used to check differences in the characteristics among the certified and non-certified children. Third, a multilevel logistic regression model (MLRM) which accounts for the binary nature of the outcome variable and the hierarchical structure of the data, was employed to analyse the relationship between the SED factors and birth certification. The MLRM which accomodates both fixed and random effects helps prevent misleading conclusions about the relative importance of the different sampling levels on the child’s BC [38]. Further, a multilevel poisson model [MPM] was used to test the sensitivity of the MLRM estimates. Studies suggest that the poisson regression can be adapted for dichotomous variable when the prevalence is no longer rare e.g., larger than 10%, as in such cases the differences between the odds ratio and the relative risk may not be negligible [39].

In the MLRM analysis, the children *i* are nested within communities *j.*. The two-level MLRM which follows the Bernoulli (*π*_*ij*_) distribution with a logit link function is defined as follows:

1 $${BC}_{ij}={\beta}_{0,0}^{\ast }+{\sum}_{a=1}^m{\beta}_{a,0}^{\ast }{W}_{a, ij}+{\sum}_{b=1}^p{\beta}_{b,0}^{\ast }{X}_j+{\varepsilon}_{0,j}$$

where *BC*_*ij*_ is the predicted log odds of individual child *i* (Level-1) living in community *j*, (Level-2) having their birth certified. ${\beta}_{0,0}^{\ast }$ denotes the overall intercept (the grand mean of Level-2), ${\beta}_{a,0}^{\ast }$ and ${\beta}_{b,0}^{\ast }$ are respectively vectors containing the *m*th and *n*th coefficients associated with W (Level-1) and X (Level-2) predictors of BC. Further, *ε*_0, *j*_ represents the random effect at the community-level - variance between communities - based on an assumption that *ε*_0, *j*_ ~ *N*(0, ${\sigma}_{com}^2$) follows a normal distribution with mean zero and the covariance matrix for a two-level model. The logistic transformation for Eq. (1) is as follows:

2 $${BC}_{ij}=\ln \left(\frac{\pi_{ij}}{1-{\pi}_{ij}}\right)$$

and represents the probability that an *i*th child in the *j*th community (com) will have their births certified. A positive regression estimate indicates a positive association between the factors and BC, and vice versa. Four separate models were fitted for both the MLRM and the MPM, respectively: *Model 1 (null model) was* fitted without any explanatory variables to measure the variation across communities and estimate the intraclass correlation coefficient (ICC) at the community level. The ICC measures the correlation between two observations within the same community i.e., the proportion of total observed individual variation in BC attributed to variations between communities. The larger the ICC (i.e., the higher the correlation *within* the clusters), the lower the variability is *within* the clusters, and consequently, the higher the variability is *between* the clusters [40]. The second model was adjusted with the child-, parent- and household-level characteristics; the third model was adjusted for the community-level characteristics only, while the fourth model adjusted for all the explanatory variables. Year-and-month dummies for the interview date and the child’s birth were also included in all models, except the null model, to account for the differences in lag lengths, and potential observed and unobserved heterogeneity in trends across space and time [41].

The beta (β) estimates are the FEs of the MLRM, and capture the overall average relationship (*measures of association*) between BC and the predictor variables. They are expressed as adjusted odds ratios (AORs) with their corresponding 95% confidence intervals, and a *P*-value of <0.05 was used to declare a statistically significant association between the variable and BC. Further, the intercepts and residual errors represent the RE and imply how the relationships between the communities differ from the overall average relationship (*measures of variation*). They are also assumed to be independent of the individual- and community-level characteristics, as they pertain to the random part of the model. The RE is interpreted using the ICC, the proportional change in variance (PCV) and the median odds ratio (MOR). In an MLRM, the individual level (Level-1) has a standard logistic distribution with variance $\frac{\pi^2}{3}$ ≈ 3.29.(^12^ π denotes the mathematical constant 3.1416 and not the probability.) The ICC is the correlation between two children within the same community (explains cluster variation), and is calculated as ${ICC}_{com}=\frac{\tau^2}{\tau^2+\frac{\pi^2}{3}}$; *τ*^2^ is the estimated variance of communities/clusters and $\frac{\pi^2}{3}$ is the children/individuals’ variance.(^13^ The ICC is calculated based on the widely adopted latent response formulation that assumes a latent continuous response underlies the observed binary response.) *ICC*_*com*_ is used to evaluate whether the community factors are more relevant in explaining the variation in the outcome variable and establish the need for the multilevel analysis [38, 40]. The higher the ICC, the larger the contribution of the community factors in explaining the variation, and an ICC of less than 5% at the null model suggests that hierarchical modelling may not be necessary [42]. The PCV, which indicates the additional effects of the included variables (individual- and community-level factors), is calculated as [PCV = $\frac{V_e-{V}_m}{V_e}$], where *V*_*e*_ is the variance in the null model and *V*_*m*_ is the variance in the successive models. Finally, the MOR is defined as the median value of the odds ratio between the highest risk area and the lowest risk area when two areas are randomly sampled and is calculated as [MOR = exp.($\sqrt{2\ast {\sigma}_{\mu}^2\ast {\phi}^{-1}(0.75)}$], where ${\sigma}_{\mu}^2$ is the cluster variance and *ϕ*^−1^ is the inverse cumulative standard normal distribution function which is approximately 0.6745. In simple terms, the MOR is a measure of unexplained cluster heterogeneity and can be interpreted as the median increased odds of being certified when a child moves from an area with a low to an area with a higher probability of certification. The higher the MOR is, the larger the general contextual effect. If the MOR is equal to 1 (i.e., ${\sigma}_{\mu}^2$ = 0), it implies the absence of a neighbourhood variation.

It is important to note at this point that the non-numerical-based categorical variables - gender, size-at-birth, religion, ethnicity, and place of residence - are *effect-coded* for the regressions, which is coded to yield a sum to zero constraint [43]: in the case of two distinct values (e.g. gender) this will have values of 1 and − 1. This is especially important for the MLRM as effect-coded variables have greater speed of convergence than dummy-coded variables [43]. All regressions were conducted in Stata 16 MP [44]. To further accommodate the complex nature of the NDHS, the data was adjusted for under-reporting using the “*svyset”* command prior to statistical analysis and the *svy* command was used to run the commands. The data were weighted using sampling weights adjusted for the unequal probability of selection between the geographically defined strata as well as the non-responses to ensure that the findings can be generalized [45]. Given that the final sample is based on a pooling three rounds of the NDHS, the sampling weight provided in the data have been de-normalized and scaled. A detailed explaination of the procedure for de-normalization and re-scaling can be found in the DHS methodology reports [45].

## Results

### State-level prevalence rate of birth certification in Nigeria

**Fig. 1:**
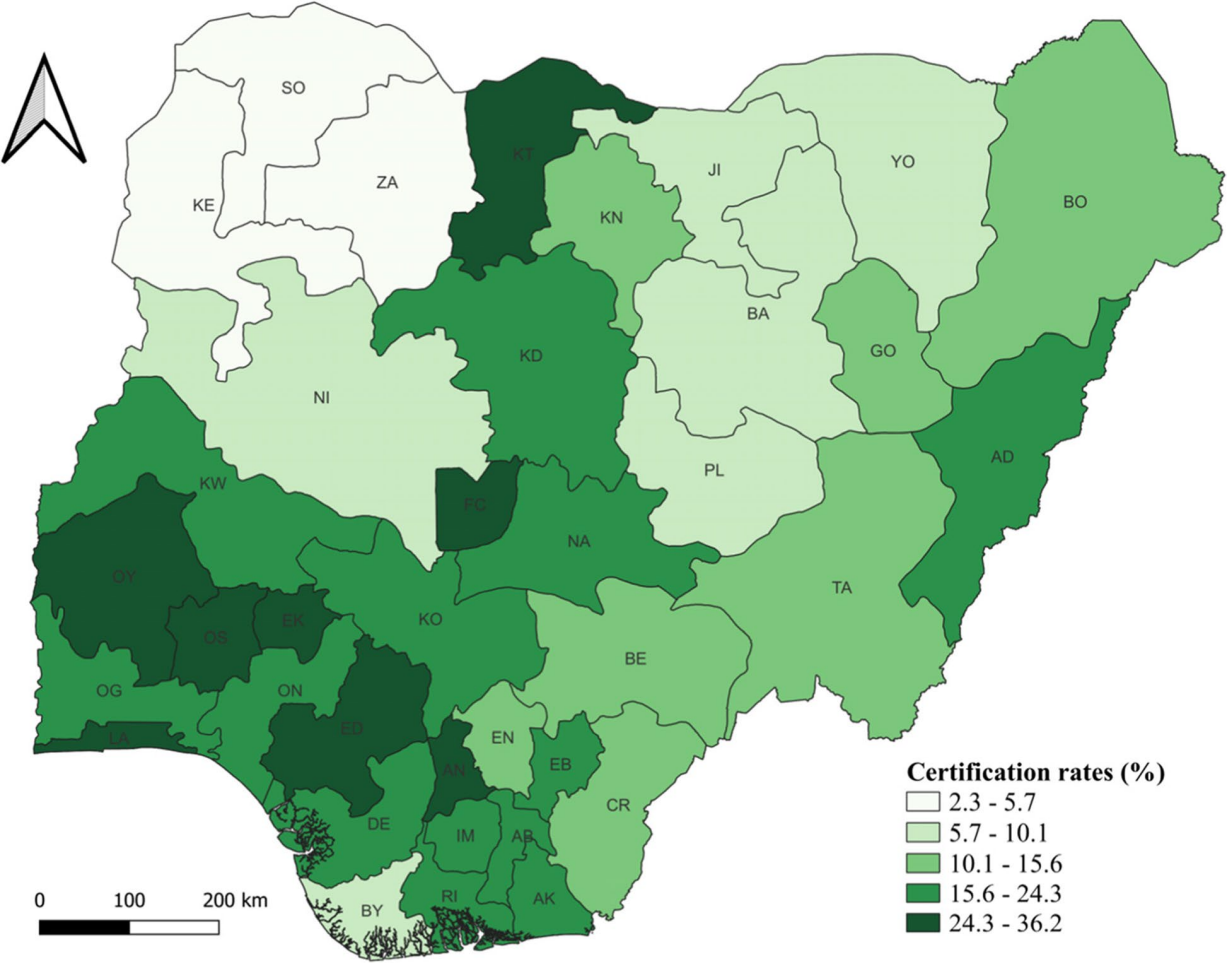
Map of Nigeria showing the birth certification rates of children under-5 years across the 37 administrative states. Note: AB - Abia, AD - Adamawa, AK - Akwa Ibom, AN - Anambra, BA - Bauchi, BY - Bayelsa, BE - Benue, BO - Borno, CR - Cross River, DE - Delta, EB - Ebonyi, ED - Edo, EK - Ekiti, EN - Enugu, FC - Federal Capital Territory, GO - Gombe, IM - Imo, JI - Jigawa, KD - Kaduna, KN - Kano, KT - Katsina, KE - Kebbi, KO - Kogi, KW - Kwara, LA - Lagos, NA - Nasarawa, NI - Niger, OG - Ogun, ON - Ondo, OS - Osun, OY - Oyo, PL - Plateau, RI - Rivers, SO - Sokoto, TA - Taraba, YO - Yobe, ZA - Zamfara

Fig. 1 presents the distribution of the proportion of birth certification of children under-5 years across the 37 administrative states in Nigeria and suggests variation across states. Zamfara (ZA) state had the lowest proportion of children under-5 years who had their births certified (2.3%, CI: 0.93–3.73), and the Federal Capital Territory (FCT) in the NC region recorded the highest proportion (36.2%, CI: 31.16–41.31). Besides the FCT, only four states have BC coverage of 30% or more - Katsina (NW, 30.00%, CI: 23.96–36.05)), Lagos (SW, 30.98%, CI: 27.72–34.24), Anambra (SE, 32.70%, 27.44–37.96), and Oyo (SW, 33.43, CI: 27.80–41.07). Among the 10 worst-performing states, the north accounts for 90% of the states, and the NW accounts for 50% of the states in the group. Following Zamfara state in order of rank is Sokoto (NW, 3.12%, CI: 1.71–4.52), Kebbi (NW, 5.74%, CI: 3.79–7.68), Niger (NC, 7.72%, CI: 5.38–10.07), Bauchi (NE, 8.02%, CI: 5.44–10.59), Bayelsa (SS, 8.66%, CI: 5.49–11.83), Yobe (NE, 8.81%, CI: 5.16–12.45), Jigawa (NW, 9.28%, CI: 6.10–12.45), Plateau (NC, 10.07%, CI: 7.26–12.89), and Kano (NW, 12.66%, CI: 9.68–15.65).(^14^ For more details, see Section D of the additional data file.) The North-South gap is not surprising, as it has been documented in various studies for a wide range of development outcomes [46–48].

### Univariate analysis of proportion and associations between predictors and birth certification

#### Individual-level SED characteristics

Table 1 provides a description of the SED characteristics described above under child-, parental-, household-, and community-level factors, respectively (Column 3). A total of 66,630 children under-five years were included in the analysis. Of these, 17.1% (*n* = 11,072; 95% CI: 16.3–17.9) had their births certified, which implies that 83% are at risk of not being recognized by the legal system of Nigeria. Splitting the sample reveals that the certification rates have been growing over time. On average, 12.1% of children under-five years had their births registered in 2008 compared to 15.8% in 2013 and 23.4% in 2018. Slightly more than half of the children were male (50.8%). The most and least populated age groups are the youngest children (0–11 months) and oldest children (48–59 months) who make up 22.9 and 17.0% of the sample, respectively. It is common knowledge that Nigerian women have more children ever born than the global average at 2.5 births [49]. This is evident in the data, as children in the 4th- or higher-order birth category make up on average 48.5% of the sample. A small proportion of children are reported to have been small at birth (13.6%) and more than half of the sample have a birth interval of ≤2.5 years (53.4%). The size at birth variable is included in the analysis due to the suggestions in the literature that parental investments can be responsive to the initial child health endowments [50]. Finally, having an SBA and being vaccinated were included to capture other forms of parental investments into the child’s development. A large proportion of children (63.7%) had received at least one of the recommended vaccinations; however, only 36.7% of the children had access to an SBA.

**Table 1:** Socioeconomic and Demographic Characteristics of the Sample, NDHS 2008–2018 (*n* = 66,630)

				Birth is certified				
Variables	Categories	Total		No		Yes		
		***N***	(%)	***n***	%	***n***	(%)	χ2
Birth is certified	No	55,558	82.9					
	Yes	11,072	17.1					
**(a) Child characteristics**								
Gender	Female	32,778	49.2	27,335	49.3	5403	48.8	
	Male	33,852	50.8	28,223	50.7	5669	51.2	0.814
Age (months)	0–11	15,249	22.9	12,723	23.4	2214	20.0	
	12–23	13,839	20.8	11,556	20.8	2314	20.9	
	24–35	12,890	19.3	10,723	18.9	2314	20.9	
	36–47	13,316	20.0	11,112	19.7	2369	21.4	
	48–59	11,336	17.0	9445	17.1	1849	16.7	81.111***
Birth order	1st	11,480	17.7	9834	17.0	2358	21.3	
	2nd	11,810	18.0	10,000	17.3	2347	21.2	
	3rd	10,524	15.9	8834	15.7	1838	16.6	
	4th or higher	32,816	48.5	26,946	50.0	4517	40.8	352.732***
Size at birth	Small	9106	13.6	7556	14.3	1174	10.6	
	Average	28,950	43.2	24,001	42.7	4850	43.8	
	Large	28,574	43.2	24,001	43.0	5049	45.6	111.0124***
Birth interval	≤2.5 years	35,320	53.4	29,668	52.8	6234	56.3	
	> 2.5 years	31,310	46.6	25,890	47.2	4838	43.7	46.081***
Skilled attendant at birth	No	43,218	63.7	35,390	68.9	4241	38.3	
	Yes	23,412	36.3	20,168	31.1	6831	61.7	3813.233***
At least one vaccination	No	24,421	36.7	20,390	38.3	2945	26.6	
	Yes	42,209	63.3	35,168	61.7	8127	73.4	558.941***
**(b) Maternal characteristics**								
Age at birth (years)	< 20	7305	11.1	6167	12.0	775	7.0	360.969***
	20–24	16,568	24.8	13,778	25.0	2602	23.5	
	25–29	18,231	27.5	15,278	26.8	3543	32.0	
	30–34	12,849	19.3	10,723	18.6	2502	22.6	
	35 and above	11,677	17.3	9612	17.5	1783	16.1	
Education level	None	32,786	48.8	27,112	54.1	2558	23.1	4935.268***
	Primary	12,620	18.6	10,334	18.6	2082	18.8	
	Secondary	16,924	25.9	14,390	22.5	4739	42.8	
	Tertiary	4300	6.7	3722	4.9	1705	15.4	
Had at least 1 prenatal visit	No	21,604	32.1	17,834	36.4	1240	11.2	2758.48***
	Yes	45,026	67.9	37,724	63.6	9832	88.8	
Polygynous	No	45,708	68.9	38,279	66.9	8669	78.3	573.411***
	Yes	20,922	31.1	17,279	33.1	2403	21.7	
Occupation	Low skill	6575	9.1	5056	10.1	487	4.4	129.616***
	Medium skill	33,192	52.3	29,057	50.7	6643	60.0	
	High skill	2551	3.8	2111	3.0	864	7.8	
	Other	24,312	39.7	22,057	36.2	3078	27.8	
Decision maker	No	28,656	43.3	24,057	45.9	3421	30.9	866.859****
	Yes	37,974	56.7	31,501	54.1	7651	69.1	
Access to media	No	28,093	40.5	22,501	44.1	2558	23.1	1722.896***
	Yes	38,537	59.5	33,057	55.9	8514	76.9	
Lost 2+ children	No	44,666	67.4	37,446	65.5	8525	77.0	571.416***
	Yes	21,964	32.6	18,112	34.5	2547	23.0	
**(c) Paternal characteristics**								
Age (years)	< 25	1161	1.8	1000	1.9	122	1.1	237.602***
	25–34	18,327	27.6	15,334	27.8	2934	26.5	
	35–44	26,899	40.7	22,612	39.6	5115	46.2	
	45–54	14,425	21.3	11,834	21.7	2192	19.8	
	55 and above	5818	8.6	4778	9.1	709	6.4	
Education level	None	25,576	38.1	21,168	42.8	1716	15.5	4271.058***
	Primary	13,189	19.9	11,056	20.4	1949	17.6	
	Secondary	19,449	29.4	16,334	26.8	4683	42.3	
	Tertiary	8416	12.5	6945	10.0	2735	24.7	
Occupation	Low skill	32,097	45.3	25,168	2.1	244	2.2	1936.685***
	Medium skill	26,136	42.2	23,445	48.8	3144	28.4	
	High skill	6927	10.4	5778	40.3	5680	51.3	
	Other	1470	2.1	1167	8.8	2004	18.1	
**(d) Household characteristics**								
Owns a bank account	No	45,691	68.1	37,835	73.6	4573	41.3	4531.050***
	Yes	20,939	31.9	17,723	26.4	6499	58.7	
Wealth status	Poor	31,605	45.5	25,279	51.3	1938	17.5	5499.449***
	Average	12,853	18.7	10,389	18.9	2004	18.1	
	Rich	22,172	35.8	19,890	29.9	7130	64.4	
Religion	Islam	24,903	62.4	34,668	64.7	5669	51.2	906.698***
	Christian	40,649	36.1	20,056	33.7	5337	48.2	
	Other	1078	1.5	833	1.7	66	0.6	
Ethnicity	Hausa/Fulani	27,868	44.2	24,557	46.9	3488	31.5	2012.874***
	Igbo	7178	11.4	6334	10.2	1893	17.1	
	Yoruba	6865	11.9	6611	9.9	2392	21.6	
	Other	24,719	32.5	18,056	33.1	3299	29.8	
**(e) Community characteristics**								
Location of residence	Rural	45,341	65	36,113	30.1	6521	58.9	3444.225***
	Urban	21,289	35	19,445	69.9	4551	41.1	
Distance to registration centres	< 5 kms	30,709	49.3	27,390	44.4	8083	73	3261.703***
	5–9.99 kms	16,887	25.2	14,001	26.8	1949	17.6	
	10+ kms	19,034	25.5	14,167	28.8	1041	9.4	
Distance to roads	< 5 kms	10,832	14.6	8111	14.2	1860	16.8	371.849***
	5–9.99 kms	7120	9.4	5222	8.6	1506	13.6	
	10+ kms	48,678	76	42,224	77.3	1063	9.6	
Altitude	Low	33,239	48.9	27,168	47.9	5968	53.9	272.999***
	Medium	16,800	23.4	13,001	24.6	1938	17.5	
	High	16,591	27.7	15,390	27.5	3167	28.6	
Average wealth status	Non-Poor	33,334	52.1	28,946	46.5	8835	79.8	
	Poor	33,296	47.9	26,612	53.5	2237	20.2	4207.132***
Child mortality	No	33,333	51.6	28,668	48.1	7595	68.6	1591.030***
	Yes	33,297	48.4	26,890	51.9	3477	31.4	
Region	North Central	11,277	13.9	7723	14.3	1340	12.1	1671.389***
	North East	14,576	17.4	9667	18.4	1362	12.3	
	North West	20,793	36.3	20,168	38.0	3111	28.1	
	South East	5723	8.6	4778	8.0	1284	11.6	
	South South	6619	9.4	5222	9.0	1262	11.4	
	South West	7642	14.4	8000	12.3	2724	24.6	

Source: Authors’ estimation using NDHS data (2008, 2013, 2018). Note: The data are weighted using the Stata svy command in Stata 16. In Columns 5–7, we present results of the cross-tabulation chi-squared test between birth certification status versus the predictor variables

Consider next the characteristics of the child’s parents and household as presented in Table 1. Of the 66,630 children in the sample, 11.1% were born to adolescent mothers (<20 years). (^15^ According to UNICEF, adolescent parents are those between 10 and 19 years (see https://data.unicef.org/topic/child-health/adolescent-health/ for details).) and 48.8% were born to mothers with no formal education. About 67.9% of children were born to mothers who had least one prenatal visit while pregnant with their last child, 31.1% were born to polygynous mothers, and 56.7% were born to mothers who participated in the decision-making within the home (e.g., large purchases and social visits to family and friends). More than half of the children (59.5%) were born to mothers who had access to media (TV/Radio/Newspaper), and 32.6% were born to mothers who had lost two or more children. Of the total sample, a very small proportion of children were born to young fathers (<25 years).(^16^ According to UNICEF, young parents are those between 10 and 24 years (see https://data.unicef.org/topic/child-health/adolescent-health/ for details).

The data also suggest that only a handful of parents are engaged in high-skilled jobs (professional, technical or managerial positions). For instance, less than 4 % of mothers and approximately 10% of fathers are employed in the high-skilled sector. This is expected given the low rate of educational attainment, especially for women. At the household-level, the data shows that less than one in every three HHs has a bank account (31.9%), which implies poor access to formal institutions in Nigeria.. About 45.5% were from the poorest wealth status and 35.8% were from the richest wealth status. For religion and ethnicity, a large majority of the children are from households who adhere to the Islamic faith (36.1%) and 44.2% belong to the Hausa/Fulani ethnic group.

#### Community-level SED characteristics

A large proportion of children living in rural communities (65.0%) and more than 10 km from a road (76.0%). Conversely, only 25.5% of children live very far (10+ kms) from the registration centers. In addition, slightly less than half (48.9%) live in a low altitude community (<323 m). Further, 47.9% of the children live in poor communities, and slightly less than half of the children (48.4%) live in communities where mothers have lost at least one child. Finally, the NW region accounts for the highest proportion of the sample (36.3%) and the SE accounts for the least number of children in the sample (8.6%). The results of the cross-tabulation chi-squared test indicate that all the predictor variables in the analysis are associated with birth certification except the gender of the child which may imply that parents have no preferences for registering their children based on their gender. Thus, gender of the child is excluded from the multivariate analysis.

### Individual- and community-level determinants of birth certification of children under-five years

#### Individual-level factors

Table 2 presents the results of the fixed effects of the MLRM. A link to the healthcare system has strong effects on the likelihood of birth certification. The odds of birth certification were significantly higher among children who had an SBA [AOR = 1.283, 95% CI: 1.164–1.413] and those that had received at least one vaccination on record [AOR = 1.494, 95% CI: 1.328–1.681] compared to children without an SBA and had zero vaccinations, respectively. There is a negative association between the age of the child and the likelihood of birth certification, however the coefficient is only statistically significant for children in their fourth year of life. The odds of BC for children aged 48–59 months was lower [AOR: 0.529, 95% CI: 0.339–0.824) compared to children in their first year of life (0–11 months). Lower odds of birth certification were also observed among children of 3rd [AOR: 0.856, 95% CI: 0.765–0.959] and 4th or higher [AOR: 0.829, 95% CI: 0.730–0.942] birth orders compared to first-born children.

**Table 2:** Two-level logistic regression results on factors associated with the birth certification of children under-5 in Nigeria DHS 2008–2018, (*n* = 66,630)

Variables	Categories	Model 1	Model 2	Model 3	Model 4
**Child characteristics**					
Age	0–11		1		1
	12–23		0.895(0.769–1.042)		0.893(0.767–1.039)
	24–35		0.859(0.668–1.106)		0.851(0.661–1.096)
	36–47		0.803(0.570–1.130)		0.796(0.565–1.120)
	48–59		0.537(0.344–0.837)***		0.529(0.339–0.824)***
Birth order	1st		1		1
	2nd		0.972(0.893–1.058)		0.973(0.894–1.059)
	3rd		0.856(0.764–0.958)***		0.856(0.765–0.959)***
	4th or higher		0.830(0.731–0.943)***		0.829(0.730–0.942)***
Size-at-birth	Small		1		1
	Average		0.996(0.944–1.050)		0.994(0.942–1.049)
	Large		1.013(0.957–1.072)		1.017(0.961–1.075)
Birth Interval	≤2.5 years		1		1
	> 2.5 years		0.947(0.889–1.009)*		0.946(0.888–1.008)*
Skilled birth attendant	No		1		1
	Yes		1.304(1.184–1.435)***		1.283(1.164–1.413)***
Had at least one vaccination	No		1		1
	Yes		1.496(1.329–1.685)***		1.494(1.328–1.681)***
**Maternal Characteristics**					
Age at birth (years)	< 20		1		1
	20–24		1.245(1.087–1.427)***		1.230(1.072–1.411)***
	25–29		1.424(1.212–1.673)***		1.395(1.187–1.640)***
	30–34		1.514(1.265–1.812)***		1.479(1.236–1.772)***
	35 and above		1.453(1.191–1.774)***		1.416(1.160–1.729)***
Education level	None		1		1
	Primary		1.185(0.965–1.456)		1.168(0.952–1.433)
	Secondary		1.253(1.097–1.431)***		1.242(1.087–1.419)***
	Tertiary		1.593(1.359–1.868)***		1.559(1.329–1.829)***
Had at least 1 prenatal visit	No		1		1
	Yes		1.520(1.316–1.755)***		1.468(1.271–1.695)***
Polygynous	No		1		1
	Yes		0.900(0.807–1.004)*		0.922(0.826–1.029)
Occupation	Low skill		0.950(0.822–1.099)		1.025(0.886–1.185)
	Medium skill		1.040(0.967–1.118)		1.014(0.943–1.091)
	High skill		1.028(0.906–1.167)		1.000(0.881–1.135)
	Other		1		1
Decision maker	No		1		1
	Yes		0.988(0.900–1.085)		0.991(0.902–1.088)
Access to media	No		1		1
	Yes		1.106(0.997–1.226)*		1.100(0.992–1.219)*
Lost 2+ children	No		1		1
	Yes		0.899(0.821–0.986)**		0.908(0.828–0.995)**
**(c) Paternal Characteristics**					
Age (years)	< 25		1		1
	25–34		0.986(0.721–1.347)		0.984(0.720–1.344)
	35–44		1.041(0.755–1.437)		1.034(0.750–1.427)
	45–54		1.048(0.750–1.466)		1.035(0.739–1.448)
	55 and above		1.000(0.700–1.429)		0.981(0.685–1.403)
Education level	None		1		1
	Primary		1.024(0.762–1.375)		1.024(0.763–1.375)
	Secondary		1.338(1.161–1.542)***		1.332(1.156–1.535)***
	Tertiary		1.410(1.224–1.624)***		1.394(1.211–1.605)***
Occupation	Low skill		0.889(0.814–0.971)***		0.913(0.836–0.998)**
	Medium skill		1.004(0.926–1.088)		0.991(0.914–1.074)
	High skill		1.093(0.991–1.205)*		1.093(0.991–1.205)*
	Other		1		1
**(d) Household characteristics**					
Owns a bank account	No		1		1
	Yes		1.316(1.188–1.458)***		1.315(1.187–1.456)***
Wealth status	Poor		1		1
	Average		1.658(1.406–1.954)***		1.430(1.197–1.707)***
	Rich		2.263(1.905–1.2.688)***		1.776(1.455–2.169)***
Religion	Islam		1		1
	Christian		1.124(0.965–1.310)		1.143(0.980–1.333)*
	Other		0.743(0.565–0.977)**		0.761(0.578–1.002)*
Ethnicity	Hausa/Fulani		1		1
	Igbo		0.989(0.871–1.123)		1.087(0.929–1.270)
	Yoruba		1.207(1.077–1.353)***		1.160(1.005–1.339)**
	Other		0.885(0.813–0.964)***		0.886(0.802–0.980)**
**(d) Community Characteristics**					
Location of residence	Rural			1	1
	Urban			1.199(1.116–1.289)***	1.044(0.970–1.124)
Distance to registration centres	< 5 kms			1	1
	5–9.99 kms			0.684(0.568–0.824)***	0.827(0.687–0.995)**
	10+ kms			0.360(0.289–0.447)***	0.466(0.377–0.576)***
Distance to roads	< 5 kms			1	1
	5–9.99 kms			1.145(0.955–1.373)	1.141(0.957–1.361)
	10+ kms			0.945(0.773–1.156)	1.012(0.837–1.223)
Altitude	Low			1	1
	Medium			1.073(0.897–1.284)	1.073(0.904–1.274)
	High			1.772(1.458–2.153)***	1.583(1.310–1.913)***
Average wealth status	Non-Poor			1	1
	Poor			0.282(0.232–0.344)***	0.613(0.486–0.774)***
Child mortality	No			1	1
	Yes			0.733(0.625–0.859)***	0.917(0.786–1.070)
Region	North Central			0.943(0.826–1.077)	0.964(0.847–1.096)
	North East			1.159(0.959–1.401)	1.644(1.364–1.982)***
	North West			1	1
	South East			0.866(0.711–1.055)	0.644(0.509–0.814)***
	South South			1.021(0.869–1.199)	0.887(0.753–1.045)
	South West			1.441(1.259–1.649)***	0.984(0.829–1.167)
Constant		0.123(0.113–0.133)***	0.039(0.016–0.097)***	0.449(0.235–0.858)***	0.079(0.032–0.194)***

Notes: All regressions control for the survey year and month as well as the child’s birth year and month. Significance level: *** denotes *p* value < 0.01, ** < 0.05, and * < 0.1. Model 1 is null or empty model without any explanatory variable. Model 2 includes controls at the individual level (level one) which captures the child-, parent- and household-level characteristics. Model 3 adjusts for the community-level (level two) variables only. Model 4 is the full model and is adjusted for the individual and community level factors

Higher odds of BC were observed for all children born to non-adolescent mothers (≥20 years) and peaks for children born to mothers aged 30–34 years [AOR: 1.479, 95% CI: 1.236–1.772] compared to adolescent mothers (<20 years). Similar to the results of the SBA and vaccination, having prenatal visits increases the odds of BC: children born to mothers who had at 1 prenatal visit during their last pregnancy had greater odds [AOR: 1.468, 95% CI: 1.271–1.695], compared to those children whose mothers had no prenatal visits. Child mortality at the mother level was also significantly associated with BC: children whose mothers had lost two or more of her children had lower odds [AOR: 0.908, 95% CI: 0.828–0.995], compared to those children whose mothers had lost at most one of her children. In contrast to the insignificant effects of maternal occupation, father’s occupation is associated with the odds of BC: lower odds of BC were observed among children whose fathers were employed in a low-skilled occupation [AOR: 0.913, 95% CI: 0.836–0.998], compared to children whose fathers were not employed at the time of the survey or whose job was in the unclassified category. Parental education has a positive and significant association with the likelihood of BC. The highest odds of BC are among children born to mothers [AOR: 1.559, 95% CI: 1.329–1.829] and fathers [AOR: 1.394, 95% CI: 1.211–1.605] with tertiary education, compared to children whose mothers and fathers had no formal education.

For the household characteristics employed in the model, higher odds were observed for children living in HHs with ownership of a bank account [AOR: 1.315, 95% CI: 1.187–1.456] than children living in households without a bank account. In addition, wealth status works as a protective factor for BC: higher odds were observed for children from middle-income [AOR: 1.430, 95% CI: 1.197–1.707] and rich families [AOR: 1.776, 95% CI: 1.455–2.169], compared to children from poor families. In terms of ethnicity, the effects were mixed: higher odds were noted for children from the Yoruba tribe [AOR: 1.160, 95% CI: 1.005–1.339] and lower odds from children from other ethnicities [AOR: 0.886, 95% CI: 0.802–0.980] , compared to Hausa/Fulani children. At the community-level, access to registration centres matter for BC: lower odds were observed for children living between 5 and 9.99kms [AOR: 0.827, 95% CI: 0.687–0.995] and those living 10kms or more [AOR: 0.466, 95% CI: 0.377–0.576] from a registration centre, compared to children whose communities are less than 5kms from a registration centre. For altitude, greater odds were observed for children living in high altitude communities [AOR: 1.583, 95% CI: 1.310–1.913], compared to those living in low altitude communities.. Finally, consider the effects of the geopolitical zone where their household is located: higher odds were observed for children living in the NE [AOR: 1.644 95% CI: 1.364–1.982] and lower odds were observed for children living in the SE [AOR: 0.644, 95% CI: 0.509–0.814], compared to those living in the NW geopolitical region.

As explained above, multilevel poisson models were introduced to test the sensitivity of the estimates reported in Table 2. The results as presented in Table 3 for the fixed effects part of the two-level poisson model are qualitatively similar to the MLRM, with some estimates being overestimated by the MLRM. Among the statistically significant variables in the full model (Model 4), the results suggest several variables with a prevalence/risk ratio of at least 1.20. For instance, children at least one vaccination on record were 1.35 times [95% CI: 1.244–1.468] and those whose mothers had at least one prenatal visit were 1.36 times [95% CI: 1.223–1.503] more likely to have their births certified, than children with no vaccination or a mother who didn’t attend any prenatal visits during her last pregnancy. Further, children born to mothers who were 25–29 years, 30–34 years and 35+ years at the time of the child’s birth had a prevalence/risk ratio of 1.23 [95% CI: 1.113–1.360], 1.27 [95% CI: 1.140–1.423] and 1.25 times [95% CI: 1.103–1.413], respectively. This may suggest non-linear effects of maternal age at birth and that the highest prevalence of BC is among children born to mothers who were 30–34 at the time of the child’s birth. Parental education also plays a significant role in the likelihood of BC. Children born to tertiary educated mothers were 1.30 times [95% CI: 1.177–1.436] more likely to have their births certified, compared to those born to parents without a formal education. In addition, having a father with a secondary or tertiary education increases the likelihood of BC by 1.25 times [95% CI: 1.133–1.376] or 1.28 times [95% CI: 1.164–1.408], respectively than being born to a father without a formal education. Further, children from middle-income or rich HHs are 1.32 times [95% CI: 1.164–1.490] and 1.50 times [95% CI: 1.307–1.714] more likely to have their births certified, compared to those from poor HHs. At the community-level, children living in high altitude locations and the NE region are 1.37 times [95% CI: 1.223–1.503] and 1.39 times [95% CI: 1.223–1.503] more likely to have their births certified, compared to those from low altitude communities or from the NW region, respectively. The results also suggest factors associated with lower risk of BC, key among them are children aged 48–59 months, living 10 kms or more from a registration centre and being from the SE region. For instance, the adjusted prevalence/risk ratio for children living 10kms or more from the nearest registration center was 0.563 [95% CI: 0.479–0.662].

**Table 3:** Two-level poisson regression results on factors associated with the birth certification of children under-5 in Nigeria DHS 2008–2018, (*n* = 66,630)

Variables	Categories	Model 1	Model 2	Model 3	Model 4
		PR (95% CI)	PR (95% CI)	PR (95% CI)	PR (95% CI)
**Child characteristics**					
Age	0–11		1		1
	12–23		0.930(0.849–1.020)		0.930(0.848–1.018)
	24–35		0.904(0.777–1.051)		0.900(0.775–1.047)
	36–47		0.893(0.727–1.097)		0.890(0.725–1.092)
	48–59		0.695(0.532–0.907)***		0.690(0.529–0.899)***
Birth order	1st		1		1
	2nd		0.991(0.944–1.040)		0.992(0.945–1.041)
	3rd		0.917(0.859–0.979)***		0.917(0.859–0.979)***
	4th or higher		0.905(0.840–0.975)***		0.904(0.839–0.973)***
Size-at-birth	Small		1		1
	Average		0.996(0.964–1.029)		0.994(0.963–1.027)
	Large		1.013(0.978–1.048)		1.016(0.981–1.051)
Birth Interval	^≤^2.5 years		1		1
	> 2.5 years		0.964(0.928–1.002)*		0.964(0.928–1.001)*
Skilled birth attendant	No		1		1
	Yes		1.185(1.117–1.258)***		1.165(1.097–1.237)***
Had at least one vaccination	No		1		1
	Yes		1.356(1.247–1.474)***		1.351(1.244–1.468)***
**Maternal Characteristics**					
Age at birth (years)	< 20		1		1
	20–24		1.158(1.062–1.263)***		1.141(1.046–1.245)***
	25–29		1.258(1.138–1.391)***		1.230(1.113–1.360)***
	30–34		1.307(1.170–1.460)***		1.273(1.140–1.423)***
	35 and above		1.284(1.134–1.454)***		1.248(1.103–1.413)***
Education level	None		1		1
	Primary		1.131(0.985–1.299)*		1.111(0.968–1.274)
	Secondary		1.181(1.083–1.288)***		1.164(1.068–1.269)***
	Tertiary		1.330(1.203–1.470)***		1.300(1.177–1.436)***
Had at least 1 prenatal visit	No		1		1
	Yes		1.407(1.268–1.562)***		1.355(1.223–1.503)***
Polygynous	No		1		1
	Yes		0.929(0.867–0.994)**		0.950(0.887–1.018)
Occupation	Low skill		0.938(0.844–1.044)		1.007(0.906–1.119)
	Medium skill		1.039(0.992–1.088)		1.015(0.969–1.063)
	High skill		1.020(0.951–1.095)		0.995(0.929–1.067)
	Other		1		1
Decision maker	No		1		1
	Yes		1.006(0.949–1.067)		1.007(0.950–1.067)
Access to media	No		1		1
	Yes		1.062(0.991–1.137)*		1.055(0.986–1.130)
Lost 2+ children	No		1		1
	Yes		0.932(0.880–0.988)**		0.942(0.889–0.998)**
**Paternal Characteristics**					
Age (years)	< 25		1		1
	25–34		1.017(0.828–1.249)		1.015(0.828–1.244)
	35–44		1.048(0.849–1.293)		1.041(0.845–1.284)
	45–54		1.058(0.850–1.316)		1.045(0.840–1.300)
	55 and above		1.037(0.821–1.309)		1.016(0.805–1.283)
Education level	None		1		1
	Primary		1.026(0.827–1.274)		1.021(0.824–1.266)
	Secondary		1.258(1.140–1.387)***		1.248(1.133–1.376)***
	Tertiary		1.296(1.177–1.428)***		1.280(1.164–1.408)***
Occupation	Low skill		0.916(0.867–0.966)***		0.937(0.888–0.988)**
	Medium skill		1.007(0.960–1.056)		0.995(0.949–1.043)
	High skill		1.044(0.987–1.104)		1.047(0.990–1.107)
	Other		1		1
**Household characteristics**					
Owns a bank account	No		1		1
	Yes		1.184(1.111–1.262)***		1.185(1.112–1.263)***
Wealth status	Poor		1		1
	Average		1.536(1.368–1.724)***		1.317(1.164–1.490)***
	Rich		1.906(1.695–2.142)***		1.497(1.307–1.714)***
Religion	Islam		1		1
	Christian		1.109(0.993–1.238)*		1.117(1.002–1.246)***
	Other		0.790(0.643–0.971)**		0.807(0.659–0.988)**
Ethnicity	Hausa/Fulani		1		1
	Igbo		0.987(0.919–1.060)		1.042(0.958–1.133)
	Yoruba		1.133(1.064–1.208)***		1.089(1.005–1.179)**
	Other		0.938(0.893–0.986)**		0.945(0.892–1.001)*
**Community Characteristics**					
Location of residence	Rural			1	1
	Urban			1.137(1.081–1.197)***	1.037(0.987–1.091)
Distance to registration centres	< 5 kms			1	1
	5–9.99 kms			0.764(0.666–0.877)***	0.869(0.761–0.992)**
	10+ kms			0.465(0.392–0.552)***	0.563(0.479–0.662)***
Distance to roads	< 5 kms			1	1
	5–9.99 kms			1.099(0.969–1.247)	1.095(0.973–1.232)
	10+ kms			0.938(0.814–1.081)	0.986(0.867–1.122)
Altitude	Low			1	1
	Medium			1.042(0.916–1.186)	1.042(0.925–1.173)
	High			1.508(1.308–1.739)***	1.374(1.202–1.571)***
Average wealth status	Non-Poor			1	1
	Poor			0.385(0.332–0.446)***	0.678(0.573–0.802)***
Child mortality	No			1	1
	Yes			0.785(0.697–0.883)***	0.916(0.822–1.022)
Region	North Central			0.962(0.873–1.062)	0.966(0.884–1.056)
	North East			1.127(0.980–1.296)*	1.391(1.224–1.582)***
	North West			1	1
	South East			0.916(0.797–1.051)	0.768(0.664–0.888)***
	South South			1.016(0.906–1.139)	0.914(0.819–1.020)
	South West			1.283(1.172–1.404)***	1.010(0.910–1.121)
Constant		0.105(0.098–0.113)***	0.035(0.019–0.062)***	0.251(0.162–0.389)***	0.064(0.036–0.115)***

Notes: All regressions control for the survey year and month as well as the child’s birth year and month. Significance level: *** denotes *p* value < 0.01, ** < 0.05, and * < 0.1. Model 1 is null or empty model without any explanatory variable. Model 2 includes controls at the individual level (level one) which captures the child-, parent- and household-level characteristics. Model 3 adjusts for the community-level (level two) variables only. Model 4 is the full model and is adjusted for the individual and community level factors

### Random effects

Table 4 presents the results of the measures of variation (REs) from the MLRM and the two-level poisson model. The null model showed that there was a statistically significant variability in the odds of BC across communities (*τ* = 3.249, 95% CI: 2.969–3.556). After controlling for all the predictor variables, Model 4 shows that the between-cluster variability (ICC) declined to 31.6%. This value is still significant which indicates that the assumption of independent observation was violated, justifying the use of multilevel analysis. For the final model, the PCV suggests that the individual and community-level factors accounted for about 53.28% of the variation observed for BC in Nigeria. In terms of the median odds ratio, the null model suggests that if a child moved to another community with a higher probability of certification, the median increase in the reference child’s odds of certification would be almost six-fold (MOR = 5.6). The median rate ratio (MRR) suggests a more conservative value of 3.39, which indicates that the level of clustering is 3.39 times higher than the reference (MRR = 1). The unexplained community variation in birth certification decreased to MOR of 3.24 and MRR of 2.10 when all factors were controlled for, which still suggests significant clustering in the full model. They imply that if a child moves from one community to a better community, the median increase in the risk of BC could be at least two-fold. The MOR and MRR suggest that when all factors are considered (full model), the effect of clustering is still statistically significant.

**Table 4:** Regression results for the three-level model of birth certification (measures of variation)

	Model 1^a^	Model 2^b^	Model 3^c^	Model 4^d^
**Panel A: Multilevel Logistic Regression Model**				
Variance τ (95% CI)	3.249 (2.969–3.556)	1.614 (1.451–1.796)	1.719 (1.553–1.905)	1.518 (1.364–1.668)
ICC (%)	49.69	32.92	34.33	31.57
PCV (%)	*Reference*	50.32	47.09	53.28
MOR	5.58	3.36	3.49	3.24
**Panel B: Multilevel Poisson Regression Model**				
Variance τ (95% CI)	1.636 (1.498–1.786)	0.639 (0.568–0.718)	0.772 (0.692–0.861)	0.608 (0.540–0.685)
PCV (%)	*Reference*	60.94	52.81	62.84
MRR	3.39	2.14	2.30	2.10

Notes: ^a^ Model 1: Null (Empty) model; ^b^ Model 2: controls for child/individual level characteristics; ^c^ Model 2: Controls for ommunity characteristics only; ^d^ Model 3: Full model; CI = Confidence Interval; ICC: Intraclass Correlation Coefficient; PCV: Proportional Change in Variance; MOR: Median Odds Ratio; MRR: Median Rate Ratio

## Discussion

This study aimed to assess the pattern of and factors associated with birth certification in Nigeria using pooled data from the 2008, 2013 and 2018 rounds of the Nigerian Demographic and Health Survey, and multiple statistical approaches. The data of 66,630 children under-five years were included in the study, and 17.1% of them had their births certified. This suggests that the nation is miles away from ensuring that children’s births are registered and certified, and this poses an obstacle to achieving SDG target 16.9 “to provide legal identity for all, including birth registration, by 2030”. The setting of Nigeria is particularly important for a study of this nature, as the country ranks high in child population, coupled with the high prevalence of poverty and low parental education (key measures of socioeconomic status). In addition, the inequality in land size across the nation also has interesting effects on access to public facilities, which in turn has significant implications for the utilization of public services. Further, the results suggest that the prevalence of birth certification was above the national average in 19 out the 37 administrative states of Nigeria, and low birth registration coverage is clustered in the northern part of the country. The finding of north-south gaps are consistent with the findings of other studies in Nigeria [46–48], and the underprovision of public facilities in northern Nigeria may have historical roots [46].

The results of the multilevel analysis suggest that individual-level characteristics may matter more than the community-level factors in explaining the patterns of BC, and several individual-level (level one) variables have strong and significant associations with BC. At level-one, access to healthcare systems (measured by the having an SBA and being vaccinated (child-level) and prenatal visits (mother-level)), maternal age at birth, ownership of bank account, and socioeconomic status (measured using parental education and wealth) are significant protective factors of BC. The positive effects of health access on BC confirm the findings of [20, 21] as well as those reported in Ghana [27] and selected Latin American countries [28], respectively. This finding reflects positive spillovers of healthcare utilization, as being closer to health services and skilled personnel increases the chances of receiving information about the need and the process of BC. The information that may be provided by healthcare officials (e.g., midwives, nurses, doctors) could help parents/caregivers decide to start the birth registration process, and complete it by collecting the child’s birth certificate from the local authorities. Maternal age at birth also increases the likelihood of BC which confirms the findings of [22–24]. However, the positive effects of maternal age at birth on BC peaks for children born to mothers aged 30–34 years and declines at advanced gestational age i.e., 35 years and above. This non-linear effect has not been noted elsewhere for BC. Other studies, however, confirm that maternal age can have a non-linear effect on child development [51, 52]. One potential explanation is that mothers at that age may have greater levels of responsibilities and time demands in-and-out of the home [53], which can lead them to postpone the certification of their child’s birth.

Evidence of a strong statistical association also exists between parental education and childhood BC: children whose parents had more years of education were more likely to be certified. This finding is in line with previous studies conducted on the determinants of birth registration in Nigeria and elsewhere [19, 28]. The arguments rest on the premise that the knowledge parents acquire from the formal education system could help them better process the information regarding step-by-step procedure for BC: receive a birth notification slip from the health centre at the time of birth and proceed to the nearest accredited centre for registration and certification of the birth. In addition, human capital theorists hypothesize that more educated parents are more likely to choose better options for their children to enhance future economic and social mobility [54]. Also, educated parents may engaged in other behaviour such as better health-seeking behaviours for their children as compared to uneducated parents which can help improve the likelihood of BC. The positive relationship between parental education and child health-seeking behaviour has been noted in Nigeria and elsewhere [55]. In addition, being from a middle-income or rich HH increases the likelihood of BC for the children, which is in line with the findings of [6, 28, 53]. Moreover, it confirms the argument that richer parents may be more aware of the importance of BC on the child’s future mobility (for example, university education, participation in the formal job market and legal migration abroad) [9]. Higher education and wealth have also been linked to better decision-making as the more educated and wealthier individuals/families are in a better position to earn money and afford the cost of registration services [56, 57]. Another interesting finding of this paper is the significant influence of bank account ownership on BC, which has not been noted anywhere else. One potential explanation is that parents/caregivers who have made contact and interact with the formal financial system may be knowledgeable about the importance of BC. For instance, one of the required documentation for opening ain bank account in Nigeria is a valid proof of age, and a birth certificate can provide such information.

Mixed results were also noted for the effect of ethnicity on the likelihood of BC: Children of Yoruba ethnic group had higher chances of being certified compared to Hausa/Fulani children. This finding is in line with the study on BC by [58]. This could be linked to the differences in cultural practices which shape the reproductive health decision-making around pregnancy, child birth and the postnatal period [59]. Notable deterrents to BC include child’s age (48–59 months), 3rd or 4th or higher-order birth (child), having two or more dead children (mother), working in a low-skilled job (father), being of minority religion (HH-level), living far away from the registration center (>5+ kms), living in a poor community and being from the SE (community-level). This finding of the negative effect of distance to registration centers on BC has not been recorded for Nigeria; however, [29] reports similar findings for children living in selected countries in Latin America and the Caribbean. The potential explanation for the negative association is that the greater distance to the registration center increases the financial and opportunity costs for the family (especially for the poor) and thus lowers the likelihood of birth registration and certification. The findings of higher birth order and father’s work status as significant obstacles to BC are also in line with other studies of birth registration [58, 60, 61]. The effect of birth order remains mixed in the literature on child development; however, a typical suggestion for the negative effect on BC is related to the delay in the benefits of birth registration. After the first child is born and is successfully registered and certified, without immediate returns to the certification, it becomes less likely that later-born children will have their births certified. Another explanation for the negative effect of birth order lies in the resource dilution hypothesis. Older children are proxies for larger family size, and in larger families it is hypothesized that the resources spent on caring for children are diluted and it becomes more costly for parents to make the commitment towards registration and certification [62]. Despite this, to draw any conclusions on the effects of birth order on birth certification, more empirical work needs to be done to account for unobserved heterogeneities that may occur within-families to draw causal inference. The findings from the random parts of the MLRM and the two-level poisson showed significant variance between communities even after adjusting for the characteristics at the child, parent, HH and community levels. This confirms the need for multilevel modelling techniques. This finding was consistent with the study conducted by [58] and justified by the existence of differences in coverage, social norms, cultural beliefs, geography, quality of health services and distribution of registration centers. One can thus conclude that about 31.6% of the variation in BC is attributed to differences across children nested within communities.

## Strengths and limitations

To the best of my knowledge, this paper counts among the first studies highlighting the determinants of birth certification for Nigerian children using a robust range of socioeconomic and demographic factors potentially associated with the registration and certification process. The study has numerous strengths. First, the study uses the NDHS data, the largest nationally representative and mutually comparable repeated cross-sectional data sample available for 66,630 children born to 46,672 mothers in 3127 communities in Nigeria for the period 2008–2018. The pooled NDHS sample provides substantial heterogeneity within and between communities to analyse the association between SED factors and BC. The NDHS surveys are comparable across settings due to the standardized nature of the variables within the dataset. Hence, these findings could be tested in and generalized to other developing countries for whom data are available. Second, the multilevel regression modelling corrects for bias on the parameter estimates as it explicitly models and uncovers heterogeneity in covariate effects [41]. The wide range of variables employed in the analyses allows a more realistic depiction of the socioeconomic and demographic determinants of BC for children under five years, and go beyond the small-scale studies of [19, 25, 63] and capture more variables with the potential to influence BC than [58]. This study may thus provide a guide for future empirical studies investigating the predictors of BC in sub-Saharan African context.

Despite the strengths mentioned above, it is important to note the limitations of this study. First, the cross-sectional nature of NDHS data makes it difficult to track the children over time to confirm their certification status or draw causal conclusions on the derived estimates. To verify the validity of the observed associations and make causal claims, the individual- and community-level effects need to be unpacked by using longitudinal data or instrumental variable techniques. Second, the data do not precisely state whether the children had their births certified in their current location which may affect the patterns reported at the state level. Further studies can incorporate the effect of internal migration on BC outcomes. Third, the data does not provide information on the exact timing of the certification. Hence, discussion on whether the child was registered and certified within the recommended time (i.e., within the first 60 days of birth) is outside the scope of this study. Furthermore, the spatial data used to construct the accessibility to registration centers are de-identified to ensure confidentiality of the respondents; however, this may introduce some measurement error in calculating the supply side variables. Future studies should employ longitudinal or experimental analysis to allow for a stronger generalization of the findings.

## Conclusion

Overall, this paper investigated the determinants of BC in Nigeria and contributes to the sub-Saharan and low-and-middle income context. This study has provided significant insights into the role of individual and community-level factors on the birth certification status of children under five years of age in a large sub-Saharan and middle-income country, Nigeria. The results highlight the crucial roles played by health service utilization, socioeconomic status and accessibility to registration services. The knowledge of these factors as key influencers for birth certification can help drive well-targeted policies by the government (e.g., the NPC) and local and multinational organizations interested in improving birth registration and certification rates in Nigeria. For instance, the government through the NPC can work towards improving birth certification rates by addressing geographical accessibility to registration centres. One way can be to increase the number of registration centers in the country and reduce the distance parents must travel to register and certify their children’s births (e.g., within a 5 km radius). This would need strong political will and concerted efforts from the Nigerian government and other stakeholders in the form of administrative and financial support for CRVS systems. Also, the stakeholders can design social protection programs in the spirit of the child development grant program (CDGP), with literacy components and cash transfers which are conditioned on parents registering and certifying the births of their children. Furthermore, free registration can be enforced to ensure that parents begin and complete the birth registration for the children on time. This can help improve birth certification among children born to uneducated parents and living in the poor households. The findings that children near-school going age (48–59 months) are less likely to have their births certified suggest significant delay in birth certification. This goes against the mandate of the 2003 Child Rights Act that parents should act in the best interest of the child. However, this can be addressed by large-scale campaigns on awareness on civil registration (especially child births) and its intended benefits. These can be addressed at the community level by engaging private sector and non-formal institutions (religious and community leaders, and civil society organisations) in the birth certification agenda. I suggest the design and implementation of well-targeted birth registration and health programs to ensure children are registered and certified on time. Ensuring this will be valuable to achieving the target of universal birth registration by 2030. Another suggestion is to ensure a holistic national child policy, which comprises all child development factors - health, education and protection that are necessary to ensure that children survive and thrive in adulthood. Finally, for a comprehensive policy to be enacted, it is important that future studies conduct a causal investigation for each of the significant factors found in this study.

## Supplementary Information

**Additional file 1.** Supplementary data file

## Data Availability

The dataset supporting the conclusions of this article are available in the Demographic and Health Survey (DHS) repository, https://www.dhsprogram.com/data/dataset_admin/index.cfm.

## Abbreviations

BC: Birth Certification
BR: Birth registration
FE: Fixed Effects
HH: Household
ICC: Intraclass Correlation Coefficient
MLRM: Multilevel Logistic Regression Model
MOR: Median Odds Ratio, Multilvel Poisson Model
MRR: Median rate ratio
NC: North Central
NDHS: Nigerian Demographic & Health Survey
NE: North East
NPC: National Population Commission
NW: North West
OR: Odds Ratio
PCV: Proportional Change in Variance
RE: Random Effects
SBA: Skilled Birth Attendants
SDG: Sustainable Development Goals
SE: South East
SS: South South
SW: South West.
