# Supplementary material for: Multilevel analysis of individual- and community-level determinants of birth certification of children under-5 years in Nigeria: evidence from a household survey
Source: BMC Public Health. 2022 Dec 14;22:2340. doi: 10.1186/s12889-022-14786-2 (PMC9749212; doi:10.1186/s12889-022-14786-2)
Supplement: Supplementary file 1 — Additional file 1. Supplementary data file [file 12889_2022_14786_MOESM1_ESM.docx]

# Supplementary data file

# A Definition of Variables

## Child Characteristics

**Gender** Gender is classified as *binary* and effect-coded as *female*=1 and *Male*=-1.

**Child’s age** The child’s age variable is in months, and is constructed by decoding the date-of-birth CMC provided in the NDHS data. When a reference child is *n* months, the child has completed its *nth* month of life e.g. a child who is one week old is yet to complete its 1st month of life, hence 0-month-old.

**Birth order** Birth order is defined as the child’s birth position among all the children ever born to their mother, as provided within the NDHS data.

**Size-at-birth** This is computed based on the mother’s report of the child’s weight at birth. This is used in place of the measured weight-at-birth in the health card, given that the latter is only available for very small proportion of children within the sample. Significant correlations have been found between this report and the actual weight at birth. *See* [1] for details.

**Birth Interval** This is computed using the preceding interval between births and converted to years. Given that first-born children have no elder siblings, the time from their mother’s marriage to their birth (variable v221 in the DHS dataset) is used to compute the birth interval.

**Skilled birth attendant** This is collected for all the births in the past 5 years and calculated based on the mother’s self-report of where she delivered and which health personnel attended to her during delivery. Having a skilled birth attendant is defined as whether the child’s birth was assisted by a doctor, a nurse or a midwife. This variable is a proxy for prenatal health inputs.

**Vaccination** This is computed as a binary variable if the child is reported to have received any of the nine recommended vaccinations as at the time of the survey: 1) BCG, 2) DPT1, 3) DPT2, 4) DPT3, 5) Polio0, 6) Polio1, 7) Polio2, 8) Polio3, and 9) Measles.

**Maternal Characteristics**

**Age-at-birth (years)** A continuous variable measured in years and is defined as the age of the mother at the time of the child’s birth. It has a mean of 27.7 years and a median age of 27 years at the time of the child’s birth.

**Education** This is defined on a continuous scale based on the mother’s report of her years of education of her spouse/partner. It ranges from 0 (no formal education) to 19 years of education.

**Prenatal Visits** This variable is derived from the mother’s self-report on the total number of prenatal visits during the pregnancy collected for the most recent birth in the past 5 years. It is 0 if the mother never attended a prenatal visit. According to the WHO, women should be encouraged to have prenatal (or antenatal) care visits during pregnancy at 1) 8-12, 2) 24-26, 3) 32, and 4) 36-38 weeks, respectively [2]. It is assumed that mothers with contact may be more likely to receive information on the birth registration process and thus likely to start and/or complete the process.

**Polygynous** The women are asked about their marital status at the time of the interview. Most of the women are in a union, that is, they are married or cohabiting. This variable is defined based on whether the woman is in a union and whether she is the only wife/partner of her spouse.

**Work** This is based on the mother’s self-report on the sector she works. This is converted into four groups: =1 if the mother works as an agricultural laborer, farmer, or in the unskilled manual, household or domestic sectors (low skill), =2 if the mother works in the clerical, sales, services or skilled manual sectors (medium skill), =3 if the mother works in professional/technical positions (high skill) and =4 if the mother is not working or works in an unclassified position (other).

**Decision-maker** This is defined based on the mother’s self-report on whether she is part of the decision-making process of her household, that is, she has final say or contributes to the final say on a number of items: 1) her own health care, 2) making large household purchases, 3) visits to family or relatives, and 4) deciding what to do with the money husband earns.

**Access to media** This is defined based on the report of whether the mother has access to mass media (television, radio or newspaper), where she can get information.

**Child mortality** This is calculated by counting the total number of children the mother has lost: =0 if she has lost none or one of her children and =1 if she has lost 2 or more children.

**Paternal Characteristics**

**Age(years)** This is defined on a categorical scale based on the mother’s report of her spouse/partner’s age. It ranges from 23 to 96+ years, with a mean of 39.8 and a median of 39 years. The older age of the fathers signifies large spousal gaps and high rates of polygynous marriages.

**Education (years)** This is defined on a continuous scale based on the mother’s report of the years of education of her spouse/partner. It ranges from 0 (no formal education) to 21 years of education.

The mean and median years of education are 6.4 and 6 years, respectively.

**Work** This is based on the mother’s self-report on the sector her spouse/partner works in. This is converted into three groups: =1 if the spouse/partner works as an agricultural laborer, farmer, or in the unskilled manual, household or domestic sectors (low skill), =2 if the spouse/partner works in the clerical, sales, services or skilled manual sectors (medium skill), =3 if the spouse/partner works in professional/technical positions (high skill) and =4 if the spouse/partner is not working or works in an unclassified position (other).

**Household Characteristics**

**Has a bank account** This is defined based on the household head’s report of having access to a bank account in the household: =0 if the HH does not have a bank account, =1 if the HH has a bank account.

**Wealth Index** The NDHS wealth index is computed on a continuous-scale through the principal component analysis (PCA), taking into account the household’s ownership of a diverse set of easy-to-measure assets. Components include consumer durables (e.g., radio and bicycles), housing quality (e.g., construction materials), access to facilities (e.g., electricity, water and sanitation facilities), and some country-specific attributes. We control for economic status via the wealth index, measured as the household’s quintile value on an index of wealth generated by DHS analysts applying weights to observed household assets using principal components analysis. It is converted into quintiles: =1 if the household is poorest, =2 if the household is poor, =3 if the household is average, =4 if the household is rich, and =5 if the household is the richest. In the analysis, it is reclassified into poor, average (middle-income) or rich.

**Religion** This is one of the two variables that captures the cultural practices of the household. The households are grouped into three categories before being effect-coded: =1 if the household is Muslim, =2 if the household is Christian, and =3 if the household practices other religion. After this, it is effect-coded with *Muslim*=-1 as the reference group.

**Ethnicity** This is second variable to capture the cultural practices. The households are grouped into four categories before being effect-coded: =1 if the household is Hausa/Fulani, =2 if the household is Igbo, =3 if the household is Yoruba, and =4 if the household is of the minority ethnicities in Nigeria. After this, it is effect-coded with *Hausa/Fulani*=-1 as the reference group.

## Community Characteristics

**Place of residence** This is defined based on the division of the communities within the data into rural vs urban areas. It is effect-coded: =1 if the household lives in the urban area and = -1 if the household lives in the rural area (*reference*).

**Distance to Registration center** This variable is defined as a three-group categorical variable: =1 if the household lives less than 5kms from the nearest registration center; =2 if the household lives within 5 and <10kms from the nearest registration center; and =3 if the household lives 10kms or more from the nearest registration center.

**Distance to road** This variable is defined as a three-group categorical variable: =1 if the household lives less than 5kms from the nearest major road; =2 if the household lives within 5 and <10kms from the nearest major road; and =3 if the household lives 10kms or more from the nearest major road.

**Altitude** This is to control for the remoteness of the household’s cluster. It is converted to a three-group categorical variable: =1 if the household’s community is less than 316 meters above sea level (low), =2 if the household’s community is between 316 and 443 meters above sea level (middle), and =3 if the household’s community is 443 or more meters above sea level (high).

**Proportion (%) of poor HHs** This is the proportion of poor households in the community. It ranges from 0 to 1 with a mean of 0.47 and a median of 0.44.

**Child mortality** This is defined as the community average of children mortality based on the fertility of the mothers in the sample.

**Region** This represents the six geopolitical regions in Nigeria. There are three in the North (North Central, North East and North West) and there are three in the South (South East, South South and South West).

For the purpose of the study, all continuous variables were transformed into dichotomous or categorical variables as described in the article.

# C Merging the Community-level Information with the NDHS data

The NDHS also provides GPS coordinates of the centroids of households interviewed in the survey and defines them as *clusters*. These clusters, also known as primary sampling units (PSU), are administratively defined areas and are proxies for "communities". The PSU are small and designed to be relatively homogeneous units with respect to the socioeconomic and demographic characteristics and the living conditions of the households, and consist of a part, one or more enumeration areas (EAs) within the country. They are defined at the smallest geographic units for which census data are available in Nigeria, they consist of a minimum of 50 households (NPC and ICF International (2014)). In this study, they are referred to as *communities*.

In order to ensure that the exact location of the households remain confidential, the GPS latitude/longitude coordinates provided in the spatial dataset have noise added to them using a random offset methodology which displaces them within a specified range of their actual location. For urban clusters, they are displaced within 0-2-kilometer range. For rural households, 99 percent are displaced within a 0-5-kilometer range and the remainder, 0-10 kilometers of positional error. Despite this displacement, the clusters remain within the original surveyed area. For the details on this, see <https://dhsprogram.com/What-We-Do/GPS-Data-Collection.cfm>. Using the community-level GPS coordinates provided by the NDHS, this study mapped each location to the nearest registration center and road using Stata’s *geonear* command. In all, there are 3171 communities (886 (2008), 896 (2013), and 1389 (2018)) mapped to 3641 registration centers and 4708 roads. The *geonear* program introduced by Kit Baum identifies nearest neighbors using geodetic distances (*see* <https://bit.ly/2H7wNzj> for details). The minimum distance between the nearest registration center and the nearest road was 0.1 and 0.3 kilometers, respectively. The maximum distance between the nearest registration center and the nearest road was 48.3 and 557.0, respectively. The mean and median distance to the nearest registration center were 7.6 and 5.5 kilometers, respectively. Additionally, the mean and median distance to the nearest road were 150.0 and 114.7 kilometers, respectively.

1. **State-level prevalence of birth certification in Nigeria**

**Table 1** Mean birth certification rates and ranking by regions and states in Nigeria

| Region (R) | %  (95% CI) | R-rank | State (S) | %  (95% CI) | S-rank | S-mean vs. R-mean^1^ |
| --- | --- | --- | --- | --- | --- | --- |
| North-Central | 15.02  (13.43 -16.61) | 4 | Benue | 14.06 (10.37-17.76) | 24 | Lower |
|  |  |  | FCT^2^ | 36.24 (31.16-41.31) | 1 | Higher |
|  |  |  | Kwara | 20.49 (15.64-25.34) | 17 | Higher |
|  |  |  | Kogi | 19.51 (14.25-24.76) | 19 | Higher |
|  |  |  | Nasarawa | 24.25 (18.50-30.00) | 9 | Higher |
|  |  |  | Niger | 7.72 (5.38-10.07) | 34 | Lower |
|  |  |  | Plateau | 10.07 (7.26-12.89) | 29 | Lower |
| North-East | 12.32  (10.80-13.84) | 6 | Adamawa | 22.09 (17.65-26.53) | 14 | Higher |
|  |  |  | Bauchi | 8.02 (5.44-10.59) | 33 | Lower |
|  |  |  | Borno | 12.86 (8.57-17.15) | 27 | Higher |
|  |  |  | Gombe | 14.19 (10.18-18.20) | 23 | Higher |
|  |  |  | Taraba | 13.22 (9.62-16.81) | 26 | Higher |
|  |  |  | Yobe | 8.81 (5.16-12.45) | 32 | Lower |
| North-West | 13.58  (11.99-15.16) | 5 | Jigawa | 9.28 (6.10-12.45) | 30 | Lower |
|  |  |  | Kaduna | 19.10 (14.44-23.77) | 20 | Higher |
|  |  |  | Kano | 12.66 (9.68-15.65) | 28 | Lower |
|  |  |  | Katsina | 30.00 (23.96-36.05) | 5 | Higher |
|  |  |  | Kebbi | 5.74 (3.79-7.68) | 35 | Lower |
|  |  |  | Sokoto | 3.12 (1.71-4.52) | 36 | Lower |
|  |  |  | Zamfara | 2.33 (0.93-3.73) | 37 | Lower |
| South-East | 23.32  (20.78-25.86) | 2 | Abia | 18.44 (14.39-22.49) | 21 | Lower |
|  |  |  | Anambra | 32.70 (27.44-37.96) | 3 | Higher |
|  |  |  | Ebonyi | 19.90 (14.97-24.84) | 18 | Lower |
|  |  |  | Enugu | 15.57 (9.99-21.16) | 22 | Lower |
|  |  |  | Imo | 21.25 (15.49-27.00) | 16 | Lower |
| South-South | 21.21  (19.25-23.18) | 3 | Akwa-Ibom | 21.70 (17.61-25.79) | 15 | Higher |
|  |  |  | Bayelsa | 8.66 (5.49-11.83) | 31 | Lower |
|  |  |  | Cross-River | 14.05 (9.89-18.20) | 25 | Lower |
|  |  |  | Delta | 22.81 (18.48-27.14) | 12 | Higher |
|  |  |  | Edo | 28.27 (23.02-33.52) | 7 | Higher |
|  |  |  | Rivers | 23.04 (18.30-27.77) | 11 | Higher |
| South-West | 29.28  (27.11-31.45) | 1 | Ekiti | 29.03 (22.62-35.44) | 6 | Lower |
|  |  |  | Lagos | 30.98 (27.72-34.24) | 4 | Higher |
|  |  |  | Ogun | 22.23 (17.90-26.55) | 13 | Lower |
|  |  |  | Ondo | 23.87 (18.63-29.16) | 10 | Lower |
|  |  |  | Osun | 28.06 (24.17-31.96) | 8 | Lower |
|  |  |  | Oyo | 33.43 (27.80-41.07) | 2 | Higher |
| National | 17.06  (16.25-17.87) |  |  |  |  |  |
| ***Notes*:** Author's computation from the Nigerian Demographic and Health Survey dataset (2008-2018). ^1^The mean rates are ranked at the regional and state levels, respectively. ^2^FCT: Federal Capital Territory | | | | | | |

**References**

1. McGovern ME. How much does Birth Weight Matter for Child Health in Developing Countries. Retrieved from https://scholar.harvard.edu/files/mcgovern/files/mark_mcgovern/birth_weight_may.pdf. 2016. Accessed 9 December 2017.
2. Wodon Q, Yedan, A. Obstacles to birth registration in Niger: estimates from a recent household survey. Journal of Health, Population and Nutrition. 2091;38(Suppl 1):26.
